# O.4.3-4 Associations between screen-time media use, physical activity, and positive and negative mental health outcomes among adolescents in Ireland: a cross-sectional study

**DOI:** 10.1093/eurpub/ckad133.190

**Published:** 2023-09-11

**Authors:** Chloe Forte, Stuart J H Biddle, Mats Hallgren, Catherine B Woods, Matthew P Herring

**Affiliations:** University of Limerick, Ireland; University of Bristol, UK; University of Southern Queensland, Australia; University of Jyväskylä, Finland; chloe.forte@ul.ie; Karolinska Institutet, Stockholm; University of Limerick, Ireland; University of Limerick, Ireland

## Abstract

**Purpose:**

Understanding the individual and joint associations of positive and negative mental health with screen-time and physical activity (PA) among adolescents is essential to develop enhanced guidance for prevention strategies and appropriate interventions.

**Methods:**

Participants (*n*=879, n = 463 female, mean age 14.71 (SD = 1.51) years) from second-level schools in Ireland completed a battery of well-validated questionnaires assessing hours of daily screen-time use (TV, computer, and phone), PA levels (PACE+) (low (0–2 day/week), moderate (3–4 day/week), or high (5+day/week), and mental health outcomes (anxiety (STAI-Y2) and depressive symptoms (QIDS) and positive mental health (MHC-SF)). Multiple linear regressions examined associations between screen-time, PA and mental health and one-way ANOVA’s examined differences in mental health outcomes between screen-time mode and use categories (none (0 hours), low (0.5-1.5 hours), moderate (2-4.5 hours), and high (5+ hours)). Cohen’s d effect size and 95% confidence intervals quantified the magnitude of the difference.

**Results:**

Higher computer (*β*=0.112, *p*≤0.001) and phone use (*β*=0.138, *p*≤0.001) were associated with higher depressive symptoms. Higher TV use (*β*=-0.111, *p*≤0.002) and PA levels (*β*=-0.123, *p*≤0.001) were associated with lower anxiety symptoms. Higher phone use (*β*=0.113, *p*≤0.002) and PA levels (*β*=0.116, *p*≤0.001) were associated with higher positive mental health. The magnitude of differences in depressive and anxiety symptoms across screen-time use categories were largely small-to-moderate (d = 0.02 to 0.67) and in positive mental health, ranged from small to large (d = 0.03 to 0.88). The sample was then stratified by PA level to assess the potential moderating influence of PA on the screen-time-mental health association, with mixed results.

**Conclusions:**

Results are among the first findings regarding the relationship between screen-time mode and PA levels with mental health, particularly positive mental health among adolescents in Ireland. Associations of screen-time and PA with mental health outcomes varied according to PA level and screen-time mode. The variation in these findings suggest the need to investigate the context of screen-time use and the screen-time activity engaged with. These results suggest that not all screen-time is detrimental and some, in moderation, may be beneficial for mental health. Future research should investigate longitudinal associations between screen-time, PA, and mental health.

**Support/Funding Source:**

N/A

